# Comparing Exogenous Methods to Induce Plant-Resistance Against a Bark-Feeding Insect

**DOI:** 10.3389/fpls.2021.695867

**Published:** 2021-07-20

**Authors:** Yayuan Chen, Adriana Puentes, Christer Björkman, Agnès Brosset, Helena Bylund

**Affiliations:** ^1^Department of Ecology, Swedish University of Agricultural Sciences (SLU), Uppsala, Sweden; ^2^Department of Environmental and Biological Sciences, University of Eastern Finland, Kuopio, Finland

**Keywords:** simulated herbivory, root damage, methyl jasmonate, forest regeneration, true insect herbivory, wounding

## Abstract

Exogenous application of the plant hormone methyl jasmonate (MeJA) can trigger induced plant defenses against herbivores, and has been shown to provide protection against insect herbivory in conifer seedlings. Other methods, such as mechanical damage to seedlings, can also induce plant defenses, yet few have been compared to MeJA and most studies lack subsequent herbivory feeding tests. We conducted two lab experiments to: (1) compare the efficacy of MeJA to mechanical damage treatments that could also induce seedling resistance, (2) examine if subsequent insect damage differs depending on the time since induction treatments occurred, and (3) assess if these induction methods affect plant growth. We compared Scots pine (*Pinus sylvestris*) seedlings sprayed with MeJA (10 or 15 mM) to seedlings subjected to four different mechanical bark damage treatments (two different bark wound sizes, needle-piercing damage, root damage) and previous pine weevil (*Hylobius abietis*) damage as a reference treatment. The seedlings were exposed to pine weevils 12 or 32 days after treatments (early and late exposure, hereafter), and resistance was measured as the amount of damage received by plants. At early exposure, seedlings treated with needle-piercing damage received significantly more subsequent pine weevil feeding damage than those treated with MeJA. Seedlings treated with MeJA and needle-piercing damage received 84% less and 250% more pine weevil feeding, respectively, relative to control seedlings. The other treatments did not differ statistically from control or MeJA in terms of subsequent pine weevil damage. For the late exposure group, plants in all induction treatments tended to receive less pine weevil feeding (yet this was not statistically significant) compared to control seedlings. On the other hand, MeJA significantly slowed down seedling growth relative to control and all other induction treatments. Overall, the mechanical damage treatments appeared to have no or variable effects on seedling resistance. One of the treatments, needle-piercing damage, actually increased pine weevil feeding at early exposure. These results therefore suggest that mechanical damage shows little potential as a plant protection measure to reduce feeding by a bark-chewing insect.

## Introduction

Induced plant defenses can be triggered experimentally by exogenous application of methyl jasmonate (MeJA), a hormone naturally present in plants. MeJA is a methyl ester of jasmonic acid (JA), which is involved in one of three signaling pathways mediating stress responses in plants. These pathways are (1) the octadecanoid pathway, which relies on JA, (2) the shikimate pathway which mainly involves salicylic acid (SA), and (3) the ethylene pathway, which relies on ethylene molecules (Dicke and van Poecke, 2002; Kant et al., 2015). The octadecanoid pathway is most important for defense responses following insect damage (McConn et al., 1997; Kahl et al., 2000). In particular, MeJA has been shown to be involved in several plant processes such as root growth, damage signaling, and promoting plant defenses against chewing herbivores and necrotrophic pathogens (Creelman and Mullet, 1995; Thaler et al., 2012). Given its role in defense induction, exogenous MeJA application is increasingly being proposed as a plant protection strategy against various insect pests and pathogens (Moreira et al., 2012b; Zas et al., 2014; Yu et al., 2019). Inducing defenses with MeJA prior to exposure to pests has been shown to reduce levels of damage, negatively affect herbivores and increase the likelihood of plant survival. These effects have been found to occur not only in crops such as rice and soybean (Chen et al., 2018; Senthil-Nathan, 2019), but also in conifer seedlings (Zas et al., 2014; Jiang et al., 2016). Thus, it has great potential to become a practical tool within pest management and a sustainable alternative to insecticides in conifer plant protection.

Before MeJA can be promoted as a practical plant protection measure, it is necessary to consider how the use of MeJA compares to other methods of plant defense induction. Other methods to trigger plant induced defenses include previous insect feeding and mechanical damage (Mattiacci et al., 1994); but little is known on whether these responses are comparable to those induced by MeJA (Moreira et al., 2012b). Simulated herbivory, mainly by mechanical wounding and true insect herbivory, has been shown to cause defense-related responses in several plants, e.g., *Nicotiana sylvestris*, *Pinus resinosa*, and *Arabidopsis thaliana* (Baldwin, 1988; Mattiacci et al., 1994; Lombardero et al., 2006; Herde et al., 2013), and could potentially be used as a method of induction. Moreira et al. (2012b) showed that exogenous application of MeJA, mechanical stem wounding and real herbivory by the pine weevil *Hylobius abietis*, resulted in chemical defensive responses that were quantitatively similar in Maritime pine (*Pinus pinaster*) seedlings. Likewise, insect herbivory caused chemical and anatomical changes related to increased defense in Sitka spruce (*Picea sitchensis*) (Miller et al., 2005). In some plants, these defensive responses do not only happen in the damaged area, but also in undamaged parts (Wu and Baldwin, 2009). A few other studies have investigated the role of root damage on aboveground induced defenses. For example, in a study with oilseed rape (*Brassica napus*), belowground insect herbivory or mechanical damage to roots increased the proportion of indole glucosinolates in the leaves (Griffiths et al., 1994). Indole glucosinolates are defensive compounds that accumulate after damage in e.g., *A. thaliana* and other *Brassicacea* (Agerbirk et al., 2008). In general, less is known about the defense induction effects following belowground simulated herbivory (Erb et al., 2012).

Depending on the type and strength of the damage stimulus, plants can also be primed, so that once attack happens defense responses can occur more quickly and stronger (Wilkinson et al., 2019). Thus, it has been suggested that mechanical damage and previous insect herbivory can also serve as methods of defense induction to increase protection against insect pests. Regardless of the defense induction method used, most studies have focused on the extent of defensive chemical responses following induction (Miller et al., 2005; Moreira et al., 2012a), but very few have actually examined subsequent herbivory to corroborate that the induction method indeed provides efficient protection. To determine whether a method is suitable for plant protection against insect pests, a herbivore damage test following induction treatment is necessary.

Aside from its protective effects, induction methods that enhance plant resistance (such as MeJA) can result in a negative effect on growth. This trade-off can occur because plants have limited resources to be allocated among defense, growth, development and reproduction. Thus, when a plant invests more resources in defense, it is expected that less resources are available for other purposes (Herms and Mattson, 1992). Some studies have shown that application of MeJA/JA results in fewer fruits and seeds in tomato plants, and growth reductions in young conifers (Redman et al., 2001; Gould et al., 2009; Sampedro et al., 2011). In the case of tomatoes, however, fruits from JA-treated plants were larger than those from control plants (Redman et al., 2001). Thus, even if MeJA can result in a trade-off, a loss in growth or reproduction could be compensated by other benefits such as larger fruits or increased survival in the case of conifer seedlings (Redman et al., 2001; Zas et al., 2014). The effects of other induction methods on growth are less known, thus it would be of interest to investigate how such effects compare to those of MeJA.

Here, we examined and compared the efficacy of MeJA to other plant-resistance inducing methods, i.e., various kinds of mechanical damage and true insect herbivory, in providing plant protection against a bark-chewing insect. As a model system, we used the pine weevil-conifer seedling system as different studies have shown that application of MeJA enhances resistance of conifer seedlings against this herbivore (Zas et al., 2014; Fedderwitz et al., 2016; López-Goldar et al., 2020). Moreover, a study in *Pinus pinaster* examined chemical responses following mechanical stem damage, true insect herbivory and MeJA treatment, and the results showed that these induction methods all increased chemical responses to an equivalent magnitude (Moreira et al., 2012a). It would be interesting to test whether those observed changes in defensive chemistry eventually result in less insect feeding. The pine weevil, *H. abietis* (Coleoptera: Curculionidae), is an important pest of planted conifer seedlings at regeneration sites where forest stands are clear-cut. Adult pine weevils are attracted to these sites, because they use conifer stumps as breeding substrate. If seedlings are planted during the first 3 years after clear-cutting, the parental generation and their adult offspring will feed on the stem bark of these seedlings. The feeding can cause seedling deformations, reduced growth, and high seedling mortality (Leather et al., 1999). Given increasing restrictions on the use of insecticides due to environmental and human health issues (Lalík et al., 2020), there is timely incentive to explore methods of plant protection based on plants’ intrinsic defenses and how they compare to each other.

Another factor that could be essential, yet rarely addressed in other studies, is the time interval from induction stimulus to exposure of plants to the insect pest. Various time intervals between MeJA treatment and exposure to the insect have been used in the pine weevil-conifer seedling system, with less pine weevil damage being observed a week, 1 month or even longer after MeJA treatment (Heijari et al., 2005; Sampedro et al., 2010; Fedderwitz et al., 2016; Chen et al., 2020). Several studies examining defensive chemical changes in conifer seedlings showed increased concentration of terpenes or resin 2 weeks, 4 weeks, or up to 1 month after MeJA treatment (Martin et al., 2002; Miller et al., 2005; Zas et al., 2014). Thus, time is also an important factor to consider when examining induced resistance responses after using different induction methods. For example, in a study with Loblolly pine (*Pinus taeda*), decreased resin flow was observed 1 day after wounding treatment but resin levels were higher than normal 30 days after mechanical treatment (Knebel et al., 2008). Thus, examining resistance at various time periods after induction stimulus could provide more comprehensive insight into how and when to apply these stimuli to achieve the greatest effect on plant resistance.

The purpose of the study was to investigate and compare how MeJA and other potential mechanical defense induction methods affect subsequent damage to conifer seedlings by the pine weevil. Resistance to pine weevil damage was used as a measure of the extent of induced resistance, with plants that were less damaged being considered to have experienced a greater induction following treatment. Additionally, we investigated if these effects depend on the time between induction stimulus (i.e., damage treatment) and exposure to weevils, and how the different treatments affect the growth of Scots pine seedlings. We chose two time intervals (12 and 32 days after stimulus) based on a pilot study and our previous studies on MeJA (e.g., Fedderwitz et al., 2016; Chen et al., 2020). Mechanical bark damage treatments (rectangular scars of different sizes inflicted on the stem, stem needle-piercing bark damage, and root damage) were chosen based on the types of damage that seedlings may encounter naturally, but exclude any chemical or microbial stimuli from the insect feeding. True insect herbivory was also included as a reference treatment. We intended to answer the following questions:

(1) How does bark mechanical damage and previous herbivory treatments influence the levels of pine weevil damage to seedlings, relative to treatment with MeJA?
(2) Do the effects of these treatments on pine weevil damage to seedlings differ depending on the time since induction occurred? More specifically, 12 and 32 days after treatment?
(3) How is seedling growth affected following these non-MeJA treatments relative to when MeJA is used?

## Materials and Methods

### Insect Material

To examine the induced resistance of seedlings following different treatments, we conducted two experiments where we subsequently exposed treated and control seedlings to pine weevils. The experiments were conducted in 2017 and 2020. The pine weevils used in these experiments were collected on May 27, 2017 and May 31, 2020, respectively, at the same sawmill (Balungstrands Sågverk AB, Enviken, Sweden) during their yearly migration. Before experiments, the weevils were kept in wooden rearing boxes in constant darkness at a room temperature of 10°C. Stems and branches from young Scots pine trees, and water tubes were placed inside each box; food and water were replaced once every month.

One week before the experiment, the pine weevils were brought for acclimatization from the cool dark room to the lab, where feeding tests were conducted (light-dark cycle 16 L/8 D, room temperature). The weevils were placed in plastic buckets with ventilated lids, and supplied with water and Scots pine branches. Female pine weevils were selected and placed individually in a Petri dish with a small Scots pine branch piece for 24 h. Those that fed on the branch during this period were selected and placed all together in a bucket, supplied only with water, in order to starve for 48 h before each feeding test.

#### Experiment 1: Plant Induction Treatments and Subsequent Feeding Tests at One Time Point

In order to test differences in resistance against pine weevils using different potential defense induction methods, six treatments (and undamaged controls) were applied to plants in order to trigger the induced defense of Scots pine seedlings. Since our regular nursery did not have enough seedlings, two provenances of Scots pine seedlings, known as Hade (plant height: 6–8 cm, Stora Enso Plantor AB, Sör Amsberg, Sweden) and Gotthardsberg (plant height: 7.8–13.5 cm, Stora Enso Plantor AB, Sjögränd, Sweden), were obtained from two nurseries instead. On July 17, 2017, seedlings were planted in round plastic pots (diameter: 14 cm) with commercial standard gardening soil (S-Jord, Hasselfors garden, Sweden) and kept in a greenhouse (light-dark cycle 16L/8D, temperature 20/16°C) for 1 month until the different treatments were applied. These two provenances were sown approximately at the same time of the year, and plants were 1-year-old when they were used in the experiment. However, Gotthardsberg has its origin further south in Sweden relative to Hade, and was larger in size when they were delivered to the lab.

After inflicting different potential induction treatments, area debarked by pine weevils in a feeding test was used as an inverse measure of induced resistance (seedlings receiving less damage were those considered to exhibit higher induced resistance). The following treatments were inflicted on plants 12 days before exposing them to pine weevils:

(1) Control (C): These seedlings (*n* = 34 for each provenance) received no damage at all.
(2) Methyl jasmonate treatment (MeJA): Seedlings (*n* = 34 for each provenance) were sprayed with MeJA. The concentration of MeJA (10 mM) was created by mixing MeJA (Sigma-Aldrich 95%, Ref. No. 392707) with a carrier solution of 2.5% (v:v) ethanol. Spraying was conducted once with a plastic bottle equipped with a spraying nozzle (Free-Syringe PC 1.5 liter, Jape Products AB, Hässleholm, Sweden), in a laboratory fume hood. The spraying bottle was pumped to reach the inner air pressure limit (2.5 bar) and shaken vigorously to mix the MeJA and carrier solution before each spraying occasion. Seedlings were placed in a row and the spraying nozzle was aimed horizontally at about 40 cm from the seedlings. The spraying bottle was moved manually along the seedling row, and the pots were turned 180° to spray the other side of the plants. Each seedling got approximately one second of spraying on each side, and all aboveground parts were moistened with the solution.
(3) Previous weevil feeding (WF): Seedlings (*n* = 34 for each provenance) were wounded by pine weevil feeding. One pine weevil was allowed to feed restrictively using a small custom-made cage with a transparent plastic tube (diameter: 10 mm; 25 mm long; and the top of the tube was sealed with holed plastic foil). A small opening was carved with a scalpel on each cage, to allow the pine weevil to feed on the area of the stem where the cage was attached. The cage and the pine weevil were removed when the insect had fed on about 50% of the circumference, with a vertical length of about 0.5 cm. The average scar area inflicted by the pine weevil (± standard error) was 32.2 ± 3.1 mm^2^.
(4) Piercing-needle damage to the stem bark (P): Seedlings (*n* = 34 for each provenance) were needle-pierced with a handmade tool consisting of a row of five insect pins (No. 00, diameter 0.3 mm). The five pins were fixed on a 1 cm long straight line, with a 2∼4 mm gap between pins on an eraser. With the tool, five vertical holes could be created simultaneously in the stem bark. The depth of each hole reached the xylem of the stem. Fifty holes were created by ten repeated piercings right below where the lowest needles grow, and these were evenly spread out around the stem circumference. The piercing damage area was ∼14 mm^2^ (area of each hole (0.3^2^ × 3.14) × 50 holes). This treatment imitates sap-sucking insect damage.
(5) Root bark damage (RD): Seedlings (*n* = 34 for each provenance) were wounded with a scalpel on the root bark. A rectangular scar was created on the main root bark right below the soil surface. The width of the scar was about 50% of the main root circumference, and the length was 0.5 cm. All phloem tissue was removed and the xylem was exposed within the scar. This treatment imitates damage by root-bark feeding insects such as *Hylastes* sp. beetles.
(6) Small stem window (WinS): A rectangular scar was inflicted on the stem of each individual seedling (*n* = 17 for each provenance) with a scalpel, and was located about 1 cm above the soil or right below where the lowest needles grow. The width of the scar was about 50% of the stem circumference and the vertical length was 0.5 cm. All phloem tissue was removed, and the xylem was exposed within the scar. The average scar area (± standard error) inflicted was 16.9 ± 4.2 mm^2^. This treatment imitates pine weevil damage.
(7) Large stem window (WinL): A rectangular scar was inflicted on the stem of each individual seedling (*n* = 17 for each provenance) as described for the WinS treatment above, but the vertical length of the scar was 1 cm. The average scar area (± standard error) inflicted was 23.3 ± 4.2 mm^2^. We originally intended to include only one treatment with one stem scar/window, but we considered that the wound may be too small to trigger induced resistance, and thus inflicted a larger scar on half of the seedlings (thus *n* = 17 for each stem window treatment per provenance).

Twelve days after the treatments, 288 seedlings in total were exposed to pine weevils in feeding tests. Each treatment included 48 seedlings (24 for each provenance), except treatment WinL and WinS which each included 24 seedlings (12 for each provenance). The remaining seedlings in each group were monitored for their height and diameter growth without exposure to pine weevils (see description below). Each seedling was exposed to one female pine weevil for 48 h. Potted seedlings and the corresponding pine weevil were enclosed by a plastic transparent cylinder with mesh net at the top to allow air flow, but prevent insects from escaping. After the feeding test, the number of feeding scars was recorded for each seedling, and the length and width of each feeding scar were measured using millimeter paper. Areas of all scars were added together to obtain the total stem area debarked per seedling. The number of girdled (when an entire ring of stem bark around the circumference is removed) seedlings were recorded as well. Due to limited lab space, the plant treatments and corresponding feeding tests were replicated in time and thus conducted in four consecutive rounds (two rounds per week). Each round consisted of 72 seedlings. Pine weevil individuals were not reused after each test.

A total of 120 seedlings were used to compare plant growth among treatments. For each treatment, 20 seedlings (10 for each provenance) were kept in a greenhouse for growth measurements [except treatment WinL and WinS which each included 10 seedlings (5 for each provenance)]. The settings in the greenhouse were 16 h light/8 h dark, and day/night temperature was 20/16°C. The aboveground height and basal diameter of seedlings was measured once a week for three consecutive weeks from August 15 to September 4, 2017. The first measurements of height and diameter were conducted 1 day before the different treatments were applied. The average height (± standard error) and diameter (± standard error) of the provenance Hade (height: 14.82 ± 0.24 cm; diameter: 2.18 ± 0.04 mm) was significantly lower than that of Gotthardsberg (height: 21.92 ± 0.47 cm; diameter: 2.46 ± 0.04 mm).

#### Experiment 2: Plant Induction Treatments and Subsequent Feeding Tests at Two Time Points

To further investigate if Scots pine resistance to weevils differed depending on the time since induction treatments were applied, we conducted a follow-up experiment. In this experiment, we examined five induction methods (in addition to non-damaged controls) and evaluated their effect on seedling resistance to pine weevil damage at two time points after treatment, 12 and 32 days. We included needle-piercing (P) of the stem bark and stem window damage (treatments P and WinS in experiment 1, respectively; average scar area Win S ± standard error: 17.8 ± 3.3 mm^2^), previous pine weevil feeding (WF in experiment 1; average scar area ± standard error: 29.0 ± 3.7 mm^2^), MeJA treatment (two levels: 10 mM and 15 mM) and an undamaged control group (C). The greenhouse settings and experimental set-up were the same as for experiment 1, except that only seedlings of the Hade provenance (average height: 13.21 ± 0.13 cm, diameter: 2.36 ± 0.02 mm; Stora Enso Plantor AB, Sör Amsberg, Sweden) were used. On July 16, 2020 (roughly a month earlier in the season relative to when experiment 1 was conducted), damage treatments were inflicted after seedlings had grown for 28 days in the greenhouse (planted on June 18, 2020). Seedlings were kept in this same greenhouse until it was time to expose them to pine weevils and evaluate induced resistance. The pine weevil exposure feeding tests were conducted 12 days after damage treatments (July 29, 2020, referred to as early exposure hereafter) for 5 of the treatments (8 seedlings × 5 treatments: C, 10 mM MeJA, 15 mM MeJA, WinS and Piercing), and 32 days after damage treatments (August 17, 2020, referred to as late exposure hereafter) for all six treatments (12 seedlings × 6 treatments: C, WF, 10 mM MeJA, 15 mM MeJA, WinS, and Piercing). Due to logistical challenges of restricting the amount of previous pine weevil damage (WF treatment) on seedlings and the limited number of seedlings available, we included this treatment only at late exposure. Each seedling was exposed to a starved female pine weevil for 48 h in the lab (light-dark cycle 16L/8D, room temperature), and damage inflicted was measured as described in experiment 1. Seedling height and basal diameter were measured once a week since they were planted. The room temperature during the feeding test was not recorded. However, data from the Swedish Meteorological and Hydrological Institute showed a different average air temperature for Uppsala, Sweden of 16.2 and 21.2°C, respectively, for feeding tests that happened in the early and late exposure groups.

#### Statistical Analyses

All statistical analyses were conducted in R software version 3.6.3 (R Core team, 2020) using R studio 1.2.5042 (RStudio Team, 2020), and graphs were generated using the *ggplot2* package (Wickham, 2016). Model fit was checked by visualizing residuals vs. predicted values using the *plot* command in R, and we found that models fitted well.

### Experiment 1: Plant Induction Treatments and Subsequent Feeding Tests at One Time Point

We examined the effect of all treatments on area debarked by pine weevils, by fitting a linear mixed model (*lmer* command from *lme4* package, Bates et al., 2015). The model included treatment (C, MeJA, P, RD, WF, WinL, and WinS), plant provenance (Gotthardsberg, Hade), and their interaction, as fixed explanatory variables, round (*n* = 4, due to limited laboratory space the experiment was replicated in time with a total of four consecutive rounds) as a random variable, and seedling height (measured before pine weevil exposure) as a continuous covariate. A generalized linear mixed-effects model (*glmer* command from *lme4* package) was used to analyze the effect of treatment on number of scars (family = Poisson) and girdling (family = binomial) including the same explanatory variables as for area debarked. To analyze the effect of treatment on seedling height and diameter increment, a linear model (*lm* command from the base R *stats* package, R Core team, 2020) was used. Explanatory variables included treatment, provenance, the interaction of treatment and provenance, and seedling initial height (from the beginning of the growth observation period) as a covariate. After model fitting, significance of main effects and interactions was tested with analysis of deviance using the ANOVA command from the *car* package (Fox and Weisberg, 2019). When main effects were significant (*P* < 0.05), treatment means were compared using a Dunnett’s test from the *contrast* command in the *emmeans* package (Lenth, 2020) and using treatment C and MeJA as reference levels.

### Experiment 2: Plant Defense Induction Treatments and Subsequent Feeding Tests at Two Time Points

We examined the effects of treatment and the timing of exposure to pine weevils on seedling resistance by fitting several models. A general least square model (*gls* command from the *nlme* package, Pinheiro et al., 2020), which allows for heterogeneous error variance, was fitted with area debarked and the number of feeding scars as response variables. The explanatory variables in the models were treatment (C, MeJA 10 mM and 15 mM, P, WinS), timing of exposure (12 or 32 days) and their interaction as fixed explanatory variables, and plant height as a continuous covariate. The variance function *varIdent()* in the *weights* = argument was used to specify heterogeneous error variance for the two fixed variables.

As the number of treatments was different for the two time points (early and late exposure), we also examined the effect of treatment on seedling resistance separately for each time point. To examine differences in pine weevil area debarked and number of feeding scars at early exposure, we fitted a general least square model (*gls* command from the *nlme* package, Pinheiro et al., 2020) for each variable separately. The model included treatment (C, MeJA 10 mM and 15 mM, WinS, and P) and seedling height as explanatory fixed variables, and the variance function *varIdent()* in the *weights* = argument was used to specify heterogeneous error variance for treatments. After model fitting, treatment estimated means were calculated by using *emmeans* command in the *emmeans* package (Lenth, 2020).

To examine differences in area debarked by pine weevils at late exposure, a linear model (*lm* command from the default *stats* package, R Core team, 2020) was fitted with area debarked being square-root transformed. The model included treatment (C, MeJA 10 mM and 15 mM, P, WF, WinS) and seedling height (measured before pine weevil exposure) as explanatory variables. A negative binomial generalized linear model (*glm.nb* command from the *MASS* package, Venables and Ripley, 2002) was used when analyzing the effect of treatment on number of scars, and this included the same explanatory variables as for area debarked.

The effect of treatment on seedling height increment and basal diameter increment (increment = last measurement–the first measurement for each individual) were analyzed with a linear model (*lm* command from the default *stats* package, R Core team, 2020). In this model, the explanatory variables were treatment (C, MeJA 10 mM and 15 mM, P, WF, WinS) and plant initial height as a covariate.

After model fitting, significance of main effects and interactions were tested with analysis of deviance using the ANOVA command from the *car* package (Fox and Weisberg, 2019). When main effects were significant (*P* < 0.05), differences among treatment levels were examined using *emmeans* command in the *emmeans* package (Lenth, 2020). If main effects were not significant, estimated means were still obtained using the *emmeans* command and used for plotting figures.

## Results

### Experiment 1: Plant Induction Treatments and Subsequent Feeding Tests at One Time Point

**FIGURE 1:**
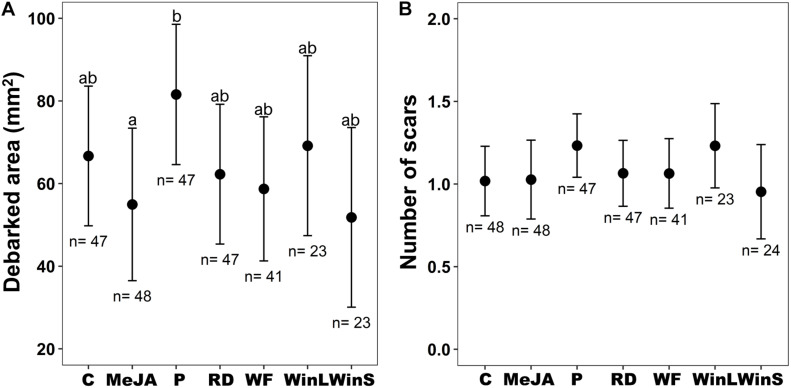
Pine weevil damage to Scots pine (*P. sylvestris*) seedlings in experiment 1 (Insect feeding tests were conducted at one time point, 12 days post-treatment) after receiving different induction treatments [Undamaged seedlings as controls (C), 10 mM MeJA (MeJA), needle-piercing damage to the stem bark (P), root bark damage (RD), previous weevil feeding damage (WF), large stem window damage (WinL), and small stem window damage (WinS)]. **(A)** Estimated mean debarked area (±95% confidence intervals), and **(B)** Estimated mean number of feeding scars (±95% confidence intervals) for each treatment. Sample sizes (*n*) used in the statistical analyses are also shown. Different letters above means indicate statistically significant differences (*P* < 0.05) between mean estimates.

Area debarked by pine weevils did not differ among Scots pine seedlings exposed to different induction treatments (Table 1 and Figure 1A), and these effects were consistent for the two seedling provenances examined (non-significant treatment × provenance interaction, Table 1 and Supplementary Figures 1A,B). Likewise, the number of pine weevil feeding scars was not significantly affected by induction treatments (Table 1 and Figure 1B). However, a significant interaction between treatment and provenance with respect to the number of feeding scars was found, with Hade receiving overall fewer scars (Supplementary Figures 1C,D). Moreover, Hade showed a significantly higher girdling rate than Gotthardsberg (21% vs. 14% of seedlings were girdled, respectively) in the feeding test (Table 1 and Supplementary Figure 2). Seedlings in the piercing treatment were 22% more debarked in area than control seedlings, and significantly more debarked than seedlings with MeJA treatment (Dunnett’s test, df = 248, *t*-ratio = 2.68, *P* = 0.040). All seedlings receiving previous pine weevil feeding (WF), and mechanical stem windows (WinL and WinS) were fed upon by pine weevils in the feeding tests, while a few seedlings from other treatments remained undamaged (Supplementary Figure 2). Moreover, previous pine weevil feeding (WF) and small stem window (WinS) resulted in similar levels of debarked area and number of scars compared to seedlings in the MeJA treatment (Figures 1A,B). In addition, seedlings in the MeJA, WF, and WinS treatments experienced reductions in area debarked of 18, 12, and 22%, respectively, compared to controls. Among the three treatments for which seedlings experienced slightly less debarked area compared to controls, seedlings with previous pine weevil feeding (WF) were more girdled than those in the MeJA and WinS treatments (girdling rate 37, 13, and 13% respectively) (Supplementary Figure 2).

**TABLE 1:** Results of analysis of deviance (df: degrees of freedom; *χ*^2^: Chi-square value; LR *χ*^2^: likelihood ratio Chi-square value; *P*: *P* value) from several models examining the effect of treatment on subsequent pine weevil damage in experiment 1.

	Debarked area			Number of feeding scars			Girdling rate		
Source of variance	df	χ^2^	*P*	df	χ^2^	*P*	df	LR χ^2^	*P*
Treatment	6	11.32	0.08	6	4.75	0.58	6	6.40	0.38
Provenance	1	6.38	**0.01**	1	26.28	**<0.01**	1	5.30	**0.02**
Height	1	2.23	0.15	1	0.41	0.52	1	0.10	0.75
Treatment × Provenance	6 × 1	8.23	0.22	6 × 1	20.04	**<0.01**	6 × 1	5.92	0.43

*More specifically, these models examined the effect of different plant defense induction treatments [Large stem window damage (WinL), small stem window damage (WinS), needle-piercing damage to the stem bark (P), root bark damage (RD), previous weevil feeding damage (WF), 10 mM MeJA (MeJA), and undamaged seedlings as controls (C)] on levels of damage (area debarked, mm^2^), number of feeding scars, and girdling rate by pine weevils (*H. abietis*) in Scots pine (*P. sylvestris*) seedlings for experiment 1 (Insect feeding tests were conducted at one time point, 12 days post-treatment). The models included the fixed variables: treatment, provenance (Hade, Gotthardsberg), their interaction, and seedling height (cm, measured a day before feeding test) as a continuous covariate. Significant effects (*P* < 0.05) are in bold.*

**FIGURE 2:**
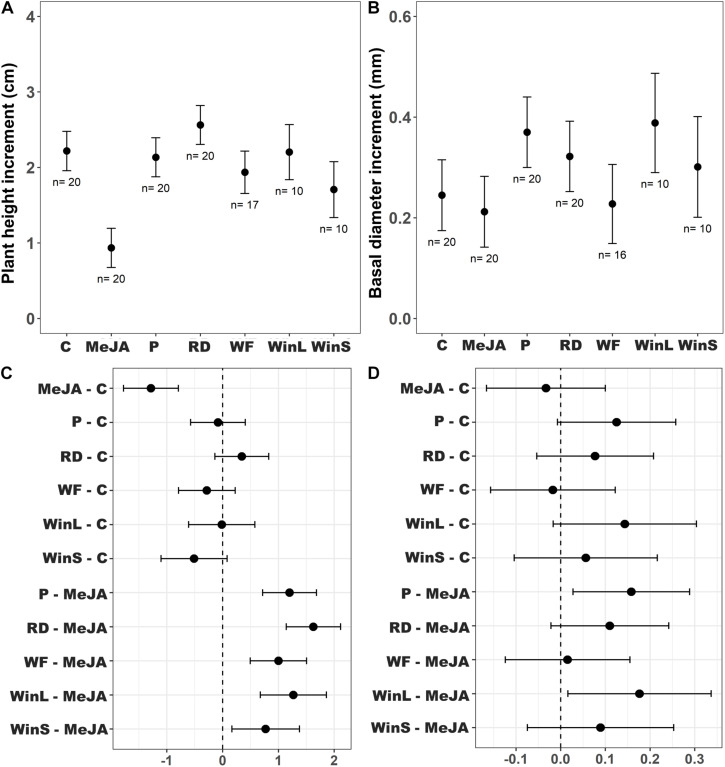
Growth increment of Scots pine (*P. sylvestris*) seedlings and treatment mean comparisons using Dunnett’s test among different plant defense induction treatments [Undamaged seedlings as controls (C), 10 mM MeJA (MeJA), needle-piercing damage to the stem bark (P), root bark damage (RD), previous weevil feeding damage (WF), large stem window damage (WinL), and small stem window damage (WinS)] in experiment 1. Insect feeding tests were conducted at one time point, 12 days post-treatment. The growth of seedlings was followed for 21 days post-treatment. Sample sizes (*n*) used in the statistical analyses are also shown. **(A)** Estimated mean plant height increment (cm ± 95% confidence intervals), **(B)** estimated mean basal diameter increment (mm ± 95% confidence intervals), **(C)** differences between treatments in estimated mean height increments (cm ± 95% confidence intervals), and **(D)** differences between treatments in estimated mean diameter increments (mm ± 95% confidence intervals). If an interval does not include zero, the difference between estimated means is considered to be statistically significant.

In contrast to results from feeding tests, the growth of Scots pine seedlings varied significantly among induction treatments (Table 2). Multiple comparisons indicated that MeJA treated seedlings had a significantly lower height growth than seedlings in all other treatments (Figures 2A,C). The non-significant interaction of provenance and treatment indicated that height growth patterns were similar for the two provenances across treatments (Supplementary Figures 3A,B and 4). Diameter growth was also significantly affected by the different defense induction treatments (Table 2), and it differed for the two provenances (Supplementary Figures 3C,D). Overall, seedlings treated with MeJA, and those that received root damage (RD), previous weevil feeding (WF), and small stem window (WinS) did not differ in diameter growth. Yet, seedlings receiving needle-piercing damage (P) or a large stem window (WinL) grew significantly more in diameter than MeJA treated seedlings (Figures 2B,D).

**TABLE 2:** Results of analysis of variance (ANOVA) (df: degrees of freedom; *F*: *F*-value; *P*: *P*-value) from several linear models examining the effect of treatments on plant growth in experiments 1 and 2.

		Height increment			Diameter increment		
	Source of variance	df	*F*	*P*	df	*F*	*P*
Experiment 1	Provenance	1	18.86	**<0.01**	1	0.28	0.61
	Treatment	6	15.36	**<0.01**	6	3.02	**<0.01**
	Initial height	1	5.46	**0.02**	1	14.33	**<0.01**
	Treatment × Provenance	6	1.72	0.10	6	3.28	**<0.01**
	Residuals	102			101		
Experiment 2	Treatment	5	4.63	**<0.01**	5	9.18	**<0.01**
	Initial height	1	0.22	0.64			
	Initial diameter				1	0.036	0.85
	Residuals	63			65		

*More specifically, these models examined the effect of different plant defense induction treatments [Large stem window damage (WinL), small stem window damage (WinS), needle-piercing damage to the stem bark (P), root bark damage (RD), previous weevil feeding damage (WF), 10 mM MeJA (MeJA), and undamaged seedlings as controls (C)] for experiment 1 (Insect feeding tests were conducted at one time point, 12 days post-treatment), and [Small stem window damage (WinS), needle-piercing damage to the stem bark (P), previous weevil feeding damage (WF), 10 mM MeJA, 15 mM MeJA, and undamaged seedlings as controls (C)] for experiment 2 (Insect feeding tests were conducted at two time points, 12 and 32 post-treatment) on growth (height increment, cm, and diameter increment, mm) in Scots pine (*P. sylvestris*) seedlings. The models included as explanatory variables: treatment, provenance (only included in experiment 1, Hade and Gotthardsberg), their interaction, and initial seedling height (cm) or initial seedling diameter (mm) (both were measured on the day of planting) as a continuous covariate. Significant effects (*P* < 0.05) are in bold.*

### Experiment 2: Plant Induction Treatments and Subsequent Feeding Tests at Two Time Points

**FIGURE 3:**
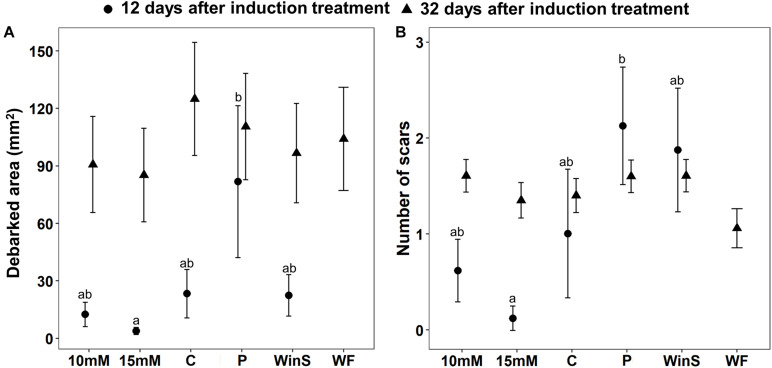
Pine weevil damage to Scots pine (*P. sylvestris*) seedlings after receiving different induction treatments [10 mM MeJA, 15 mM MeJA, undamaged seedlings as controls (C), needle-piercing damage to the stem bark (P), small stem window damage (WinS), and previous weevil feeding damage (WF, not included in the weevil exposure test conducted 12 days post-treatment)] in experiment 2. Plants that were exposed to pine weevils 12 days after induction treatments are referred to as early exposure, and those that were exposed 32 days after, are referred to as late exposure in the main text. **(A)** Estimated mean debarked area (mm^2^ ± standard error), and **(B)** Estimated mean number of feeding scars (±standard error). Sample size (*n*) is equal to 8 for all other treatments, except that the Control group (C) is equal to 7 for the early exposure, and *n* = 12 for all treatments for the late exposure. Different letters above means indicate statistically significant differences (*P* < 0.05) between mean estimates.

Pine weevil damage to Scots pine seedlings differed among treatments, and between the two time points at which seedlings were subsequently exposed to pine weevils. Overall, seedlings were significantly more damaged at late exposure, than at early exposure (Table 3). At early exposure, seedlings in the needle-piercing treatment (P) received significantly more feeding damage (both in terms of debarked area and feeding scars) than seedlings in the MeJA treatment (15 mM), and it was the only group receiving 250% more damage by pine weevils relative to control seedlings (Table 3 and Figures 3A,B). Seedlings in the MeJA treatments experienced a non-statistically significant reduction in area debarked (46% less for 10 mM, and 84% less for 15 mM) compared to control seedlings; while, seedlings in the WinS treatment received similar damage to controls (Figure 3A and Supplementary Figure 5A). Although not statistically significant, seedlings in the piercing and WinS treatments received 110 and 87% more feeding scars, respectively, than controls; while MeJA treated (10 mM MeJA and 15 mM MeJA) seedlings received 38 and 85% less scars, respectively, than controls (Figure 3B and Supplementary Figure 5B). In addition, many plants were not damaged at all at the early exposure, especially for seedlings that were treated with 15 mM MeJA, which had only one seedling damaged by pine weevil, while seedlings with needle-piercing had only one seedling undamaged. More than half of the seedlings treated with 10 mM and control seedlings were undamaged (Supplementary Figure 6A).

**TABLE 3:** Results of analysis of deviance (df: degrees of freedom; χ^2^: Chi-square value; LR χ^2^: likelihood ratio Chi-square value; *P*: *P*-value) from several models examining the effect of treatment on subsequent pine weevil damage in experiment 2.

	Source of variance	Debarked area			Number of feeding scars		
		df	F/χ^2^	*P*	df	LR χ^2^	*P*
Both time points	Time point	1	82.36	**<0.01**	1	88.83	**<0.01**
	Treatment	4	13.05	**0.01**	4	22.07	**<0.01**
	Height	1	0.36	0.55	1	1.13	0.29
	Time point × Treatment	4	2.88	0.72	4	1.24	0.87
12 days after induction treatment	Treatment	4	12.53	**0.01**	4	18.85	**<0.01**
	Height	1	0.24	0.62		0.23	0.63
32 days after induction treatment	Treatment	5	0.29	0.92	5	6.67	0.25
	Height	1	1.41	0.24	1	8.06	**<0.01**

*More specifically, these models examined the effect of different plant defense induction treatments and time point of exposure to pine weevils since induction, on area debarked (mm^2^) and number of scars in experiment 2 (Insect feeding tests were conducted at two time points, 12 and 32 days post-treatment). Models included treatments [Small stem window damage (WinS), needle-piercing damage to the stem bark (P), previous weevil feeding damage (WF), 10 mM MeJA, 15 mM MeJA and undamaged seedlings as controls (C); previous weevil feeding damage (WF) was not included at 12 days after induction], time point (12 or 32 days after induction), their interaction and plant height as a continuous covariate. Significant effects (*P* < 0.05) are in bold.*

At late exposure, area debarked and number of feeding scars did not differ significantly among treatments (Table 3 and Figures 3A,B). Seedlings in the control group, nonetheless, tended to receive the most pine weevil damage in terms of debarked area. Seedlings in the 10 mM MeJA, 15 mM MeJA, P, WF, and WinS treatments had 27, 32, 12, 17, and 23% less damage, respectively, than control seedlings (Figure 3A and Supplementary Figure 5A). The number of feeding scars was quite similar among treatments, and only seedlings with the previous pine weevil feeding (WF) and 15 mM MeJA treatments showed a 24 and 4% reduction, respectively, compared to controls. On the other hand, seedlings in the 10 mM MeJA, P, and WinS treatments received 15, 14, and 15% more scars than controls (Figure 3B and Supplementary Figure 5B). Across all treatment, the number of undamaged seedlings was lower, compared to those in the early exposure group (Supplementary Figure 6).

**FIGURE 4:**
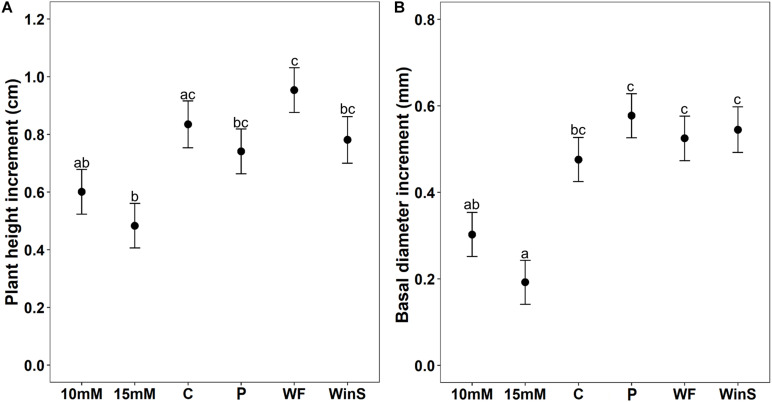
Growth increment of Scots pine (*P. sylvestris*) seedlings receiving different plant defense induction treatments [10 mM MeJA, 15 mM MeJA, undamaged seedlings as controls (C), needle-piercing damage to the stem bark (P), small stem window damage (WinS), and previous weevil feeding damage (WF)] in experiment 2 (*n* = 12 per treatment, insect feeding tests were conducted at two time points, 12 and 32 days post-treatment). The growth of seedlings was followed for 32 days post-treatment. **(A)** Estimated mean height increment (cm ± standard error), and **(B)** Estimated mean diameter growth (mm ± standard error). Different letters above means indicate statistically significant differences (*P* < 0.05) between mean estimates.

Similar to experiment 1, we found that different induction treatments significantly affected plant height and diameter growth (Table 2). Only seedlings treated with 15 mM MeJA grew significantly less (42% lower) in height than control seedlings. Seedlings treated with 10 mM MeJA and 15 mM MeJA grew significantly less (37 and 49%, respectively) in height than seedlings induced by previous pine weevil feeding (WF) (Figure 4A and Supplementary Figure 7A). For diameter growth, only seedlings treated with 15 mM MeJA grew significantly less (60%) than control seedlings. Seedlings receiving needle-piercing (P), previous pine weevil feeding (WF), and small window damage (WinS) treatments grew slightly more in diameter (21, 10, and 15% more, respectively) than control seedlings. Seedlings in these three treatments grew significantly more than seedlings treated with MeJA (10 and 15 mM) (Figure 4B and Supplementary Figure 7B). We also noted that wounds created by the different induction treatments had healed completely, or healed to at least half the original damaged area, by the time of late exposure (Supplementary Figures 8A–C).

## Discussion

Our study showed that simulated bark damage as treatments for potential plant defense induction can affect levels of pine weevil damage to Scots pine seedlings. However, these effects varied depending on the type of damage inflicted and when plants were exposed to the insects after induction stimulus occurred. We found that none of the mechanical induction methods increased seedling resistance to a greater extent than MeJA, and that a shorter time between the induction stimulus and exposure to pine weevils resulted in lower damage levels. One type of stem damage, needle-piercing, even increased subsequent feeding by pine weevils to very high levels relative to all other treatments. In terms of growth, only MeJA negatively affected seedling height growth and diameter relative to the control group, in line with previous studies. All in all, our results indicate that the previous damage treatments evaluated in this study do not provide enhanced seedling resistance to bark-feeding insect damage. We discuss our findings below.

Even though studies on other conifer plants have shown that both mechanical wounding and insect herbivory can trigger induced defensive responses (Miller et al., 2005; Moreira et al., 2012a), our bark damage treatments did not result in significantly greater seedling resistance. One explanation could be that even if defensive chemistry is enhanced or altered, these changes are not enough to sufficiently deter pine weevil feeding. Previous studies have not exposed mechanically damaged plants to subsequent insect feeding, and have assumed that increased defensive chemistry responses will result in less feeding (i.e., greater resistance). Our results show that this assumption may not always be true. Exposure to the pest after induction stimulus is essential if these methods are being evaluated for use within plant protection. Our study directly examined the extent of protection provided by previous mechanical damage, and we find that it is not sufficient against damage by a bark-chewing insect. Another factor that could also be important is the extent of damage or tissue loss. A recent study on tobacco (*Nicotiana tabacum*) plants showed that the amount of leaf tissue loss is important for the level of defense induction (Lin and Felton, 2020). The authors found higher levels of trypsin protease inhibitors (which result in anti-nutritive effects and reduced insect herbivore growth) in plants subjected to whole leaf removal relative to partial leaf damage (Lin and Felton, 2020). On the other hand, a study with 1-year old Scots pine seedlings found that moderate and severe mechanical stem damage resulted in similar negative effects on plant morphology and physiology (Bansal et al., 2013). Seedlings received either one (moderate damage) or two (severe damage) window-like stem bark scars (inflicted with a scalpel), and each scar was about 10 mm in length and covering 1/3 of the stem circumference (Bansal et al., 2013). These scars are similar and even slightly greater in total area to our WinS and WinL treatments, for which we inflicted scars of 5 or 10 mm in length, respectively, across 1/2 of the stem circumference. The authors found that both treatments significantly reduced photosynthesis, needle mass and needle area relative to undamaged controls (Bansal et al., 2013). In our study, seedlings in the WinS treatment received similar pine weevil damage to those in the MeJA treatment, and even received less damage (albeit non-statistically significant) than those with larger window wounds (experiment 1, WinS and WinL, Figure 1A). Our results and those of Bansal et al. (2013) suggest that greater tissue loss or damage may instead be detrimental for the plants, and would not necessarily result in greater enhanced resistance to subsequent insect feeding. However, evaluation of a broader range of stem damage levels may be needed to conclusively elucidate if the extent of tissue loss plays a role.

In addition to stem bark damage, root herbivory has also been shown to trigger subsequent defensive responses in aboveground plant tissues, e.g., in cotton *Gossypium herbaceum* (Bezemer et al., 2004) and tobacco *N. tabacum* (Kaplan et al., 2008b). After belowground damage occurs, it has been observed that a reduction in herbivore growth rate, body size and food consumption of aboveground herbivores can occur (Bezemer et al., 2003; Soler et al., 2005; Van Dam et al., 2005). Thus, root herbivory has the potential to decrease overall plant damage levels of aboveground herbivores. However, there are also cases where it has not resulted in increased resistance aboveground. The magnitude of defensive responses in aboveground tissue may not be large or effective enough to decrease herbivore damage, as in the case of cotton *G. herbaceum* (Bezemer et al., 2004). Moreover, some aboveground herbivores may even benefit from belowground herbivory and inflict more damage (Soler et al., 2007; Kaplan et al., 2008a). In our study, we found that root-damaged seedlings tended to grow more (15% more in seedling height increment and 33% more in basal diameter increment) and received 7% less weevil damage (debarked area) compared to control seedlings, but this difference was not statistically significant. This result suggests that the extent of root tissue loss may not have been large enough to trigger aboveground defensive responses that affected the pine weevils.

In contrast to all other induction treatments, seedlings in the needle-piercing treatment received much greater damage levels (as extreme as 250% more damage) than controls. Pine weevils are known to be attracted to the odors or compounds emitted by recently damaged seedlings (Nordlander, 1991). Given the multiple wounds that the needle piercing treatment inflicted on the stem, it could be possible that it stimulated their feeding. Even though two other treatments also inflicted large wounds (WinS and WinL), increased damage levels to the extent of those receiving piercing damage, were not observed for seedlings in these treatments. This indicates that patterns of damage are also relevant and can differentially influence pine weevil feeding behavior. However, we noted in experiment 2 that needle-piercing wounds had healed by the late exposure and at this time point, seedlings received somewhat less pine weevil damage than controls. This suggests that the cues emitted by freshly damaged seedlings could stimulate feeding, but decrease with time.

Although none of the previous damage treatments enhanced seedling resistance to a greater extent than MeJA, treatment with MeJA was also not as significantly effective as reported in previous studies. Only seedlings treated with a higher MeJA concentration (15 mM) were significantly less damaged compared to seedlings in the piercing-needle treatment after early exposure. It could be that the dose (the net amount and frequency of MeJA treatment) we used could partly explain our results. The effect of MeJA treatment on pine weevil damage has been shown to be dose dependent (Moreira et al., 2009; Zas et al., 2014). In one of our previous experiments, a higher dose of MeJA (three consecutive sprayings of 10 mM MeJA) resulted in greater Norway spruce resistance to pine weevil damage relative to plants receiving a lower dose (one spraying of 10 mM MeJA) (Chen et al., 2020). The low dose of MeJA in our previous study on Norway spruce was the same as the low dose used in this study on Scots pine seedlings, and the amount of debarked area received by these two conifer species were similar in both studies. Other studies have also used higher doses and concentrations, which have resulted in greater resistance to pine weevil damage. For example, MeJA concentrations of 100, 40, and 22 mM were used on Maritime pine (*P. pinaster*) (Moreira et al., 2009; Moreira et al., 2012a,b), 50 mM MeJA on Norway spruce (Fedderwitz et al., 2016), and 25 mM MeJA on Maritime pine, Monterrey pine (*Pinus radiata*), Scots pine and Norway spruce (Zas et al., 2014). Therefore, it appears that the MeJA dose in this study was not enough to significantly reduce pine weevil damage. Moreover, it seems that induced resistance can be better achieved by several sprayings of MeJA at lower concentrations instead of one application with a higher concentration. Concentrations higher than 10 mM can be detrimental to seedlings, and result in treatment-related damage (e.g., loss of needles, needle browning in Norway spruce; Fedderwitz et al., 2019), and we indeed observed some needle-browning in seedlings treated with 15 mM MeJA (Supplementary Figure 8D). Our results are thus, an important contribution to development of methods for optimum use MeJA as a seedling protection tool. Finding the MeJA treatment concentration and frequency that provides effective resistance, minimizes phytotoxicity and is compatible with nursery needs and practices is essential for MeJA implementation (Fedderwitz et al., 2019, Chen et al., 2020).

Timing since induction stimulus is also an essential factor in development of plant protection strategies aimed at increasing seedling resistance prior to pest exposure. We found that the effect of previous damage and MeJA on triggering seedling resistance to pine weevils differed depending on the time since treatment. Overall, we found that plants exposed early to pine weevils (12 days after treatment) received less damage relative to those in the late exposure group (32 days after treatment). These results could indicate that treatment effects were short-lasting and tended to lose their efficacy with time. As discussed in previous paragraphs, an explanation could be that the extent of tissue loss/damage could play a role and that MeJA doses used were not enough to induce effective resistance. We observed that seedlings in the 10 mM and 15 mM MeJA groups, were often not eaten by weevils at all (Supplementary Figure 6) or received considerably less pine weevil feeding damage at 12 days relative to 32 days after MeJA application. Seedlings in other induction treatments also showed a similar tendency, with less damage at 12 days relative to 32 days but not as pronounced as for those in the MeJA group. Thus, if a peak in induced resistance occurs, this peak is likely closer to 12 days rather than 32 days after treatment.

Another potential cause for the different damage levels at these two time points could be that pine weevil feeding behavior differed. The average air temperature in Uppsala, Sweden at the time when the late exposure occurred (average air temperature: 21.2°C) was 5°C higher than during the early exposure (average air temperature: 16.2°C), according to data from Swedish Meteorological and Hydrological Institute (station 97510 Uppsala Aut, 59°50′50″N, 17°37′55″E, SMHI, 2020). The weevils were acclimatized in the lab for a week before the feeding test, thus, weevils in the late exposure group might have been affected by the warmer room temperature compared to those in the early exposure group. Pine weevils have been shown to consume almost four times more bark of Scots pine twigs at 20°C compared to 15°C (Leather et al., 1994). The behavior of pine weevils may thus, have been affected by a higher room temperature and resulted in increased feeding at the late exposure time point. All in all, from a plant protection perspective, our results on the timing of induction suggest that it may be better if the treatment stimulus occurs closer rather than further from pest exposure. However, additional studies where temperature is controlled for, and different levels of tissue loss and other time points since treatment are included, would help to tease apart their effect on seedling resistance.

We were also interested in examining any potential cost of induction treatments with respect to plant growth. As documented in other studies on MeJA-induced plant defense (Heijari et al., 2005; Vivas et al., 2012), we observed a significant growth reduction in seedlings receiving MeJA treatment compared to the control seedlings. Such a trade-off between growth and defense has indeed been found for seedlings of several coniferous species, e.g., Maritime pine (Moreira et al., 2012b), Monterrey pine (Gould et al., 2008), and Norway spruce (Chen et al., 2020). Also, in line with other studies, we found that growth was even more reduced for plants receiving the higher concentration of MeJA (15 mM). This suggests that resources were diverted away from growth and presumably invested in defense, yet it only resulted in a slight reduction in area debarked relative to seedlings in the control group.

Some of the non-MeJA treatments also exhibited different relationships between growth and resistance. For example, seedlings in the piercing treatment (P) and large window (WinL) treatments received relatively higher subsequent pine weevil damage compared to all other treatments in experiment 1. Yet, there was a slight reduction in height growth and even a tendency to grow more in diameter compared to the control group. This is in line with another study that Scots pine seedlings with stem bark damage had significantly more radial growth compared to undamaged controls (Bansal et al., 2013). Moreover, seedlings with root damage showed a tendency to grow more in height, compared to control seedlings. A study on field corn *Zea mays* showed that plant dry weight was greater for plants damaged by the western corn rootworm (*Diabrotica virgifera*) relative to those not experiencing any root damage (Godfrey et al., 1993). It is also possible that the non-MeJA treatments do not induce but instead “prime” seedling defenses, which is much less costly compared to fully inducing defenses (Wilkinson et al., 2019). We observed, for example, a slight reduction in debarked area for seedlings in other non-MeJA induction methods after the late exposure, but no growth reduction compared to controls. This is in contrast to plants receiving MeJA treatments, especially at the high concentration, which exhibited distinct growth reductions, and only a reduction of 20–30% in area debarked compared to control seedlings. However, we are not able to discern from our study which of these mechanisms was involved.

## Conclusion

Our study showed that bark damage induction treatments and a low dose of MeJA did not effectively increase the resistance of Scots pine seedlings. Induction methods that include needle-piercing stem wounding can even be detrimental for seedlings, as we found that this type of damage resulted in even more damage by pine weevils relative to all other treatments. Apart from MeJA treatments, none of the damage treatments had negative effects on seedling growth in terms of height and diameter. All in all, our results suggest that mechanical damage may not be sufficient to trigger induced resistance responses that provide adequate seedling protection. Thus, these methods of induction would not be suitable for larger scale implementation to protect conifer seedlings. Instead, improving the use of MeJA and finding optimal concentrations that enhance resistance but minimize negative effects, remains as a promising alternative. Nonetheless, further studies varying the degree of tissue loss as well as the time period between induction treatment and insect exposure, would be of interest. In addition, studies that examine the levels of chemical defense in seedlings following the treatments and subsequent exposure to insect feeding in both lab and the field, are needed to enhance our knowledge on the mechanisms of induced defense in conifer seedlings.

## Data Availability Statement

The raw data supporting the conclusions of this article will be made available by the authors upon request, without undue reservation.

## Author Contributions

AP, CB, and HB conceived and designed the experiment. AB carried out the pilot experiment as part of her master’s thesis. AP, CB, HB, and YC re-evaluated and improved the experimental design. YC conducted the experiment, carried out the statistical analyses, and wrote the draft of the manuscript with input from AP, CB, and HB. All authors contributed to subsequent revisions of the manuscript and agreed to publish the final version of the manuscript.

## Conflict of Interest

The authors declare that the research was conducted in the absence of any commercial or financial relationships that could be construed as a potential conflict of interest.
