# Supplementary material for: Comparing Exogenous Methods to Induce Plant-Resistance Against a Bark-Feeding Insect
Source: Front Plant Sci. 2021 Jul 20;12:695867. doi: 10.3389/fpls.2021.695867 (PMC8329535; doi:10.3389/fpls.2021.695867)
Supplement: Supplementary file 1 [file Data_Sheet_1.docx]

Supplementary Material

# Supplementary Figures

#
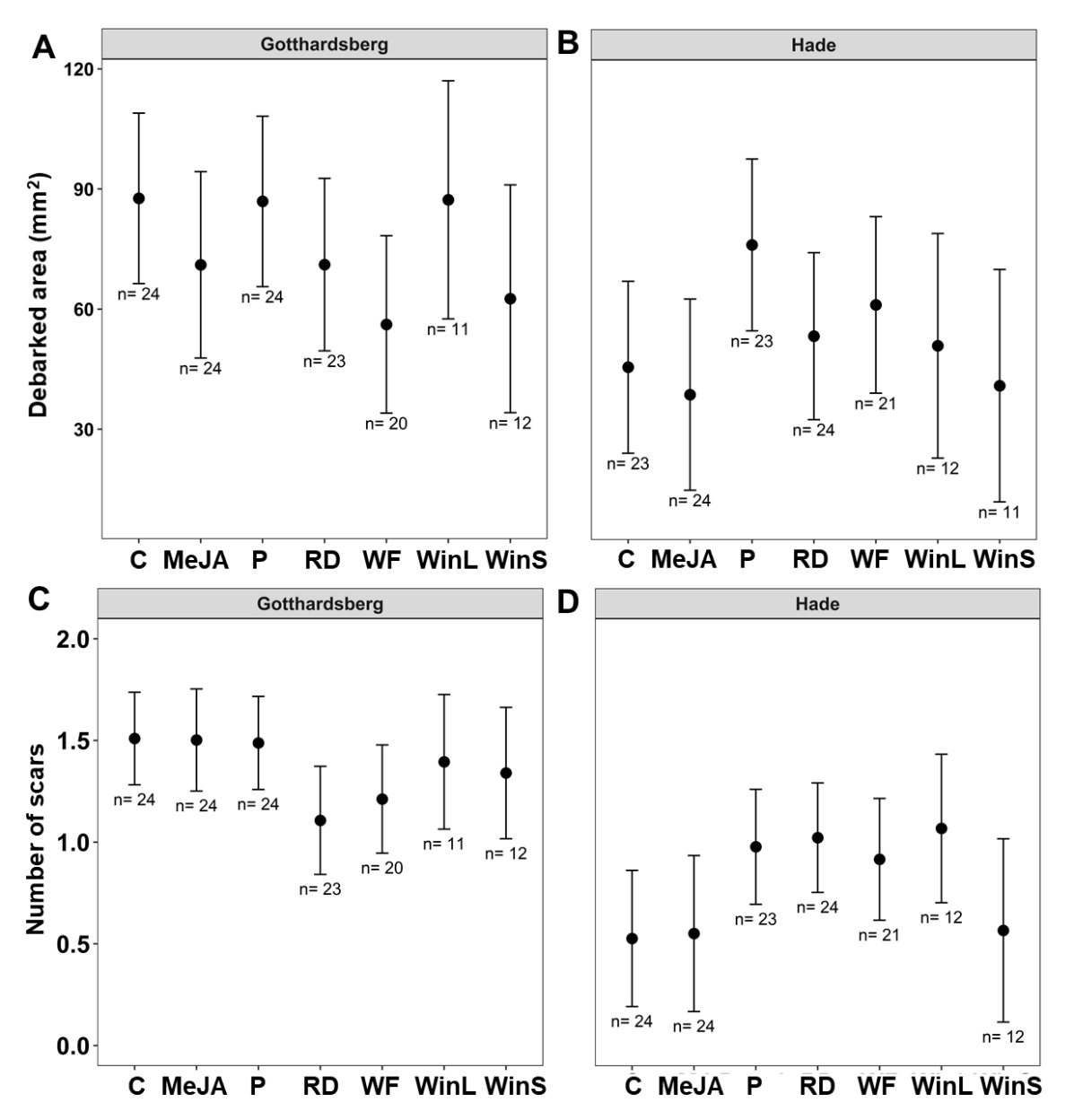


**Supplementary Figure 1.** Estimated mean debarked area (mm^2^ ± 95% confidence intervals) and number of feeding scars (± standard error) for two provenances of Scots pine (*P. sylvestris*) seedlings receiving different plant defense induction treatments (Undamaged seedlings as controls (C), 10 mM MeJA (MeJA), needle-piercing damage to the stem bark (P), root bark damage (RD), previous weevil feeding damage (WF), large stem window damage (WinL), and small stem window damage (WinS)) in experiment 1. Insect feeding tests were conducted at one time point, 12 days post-treatment. Panels **(A)** and **(C)** show the provenance Gotthardsberg, debarked area and number of scars; panels **(B)** and **(D)** show provenance Hade, debarked area and number of scars. Sample sizes (n) used in the statistical analysis are also shown.


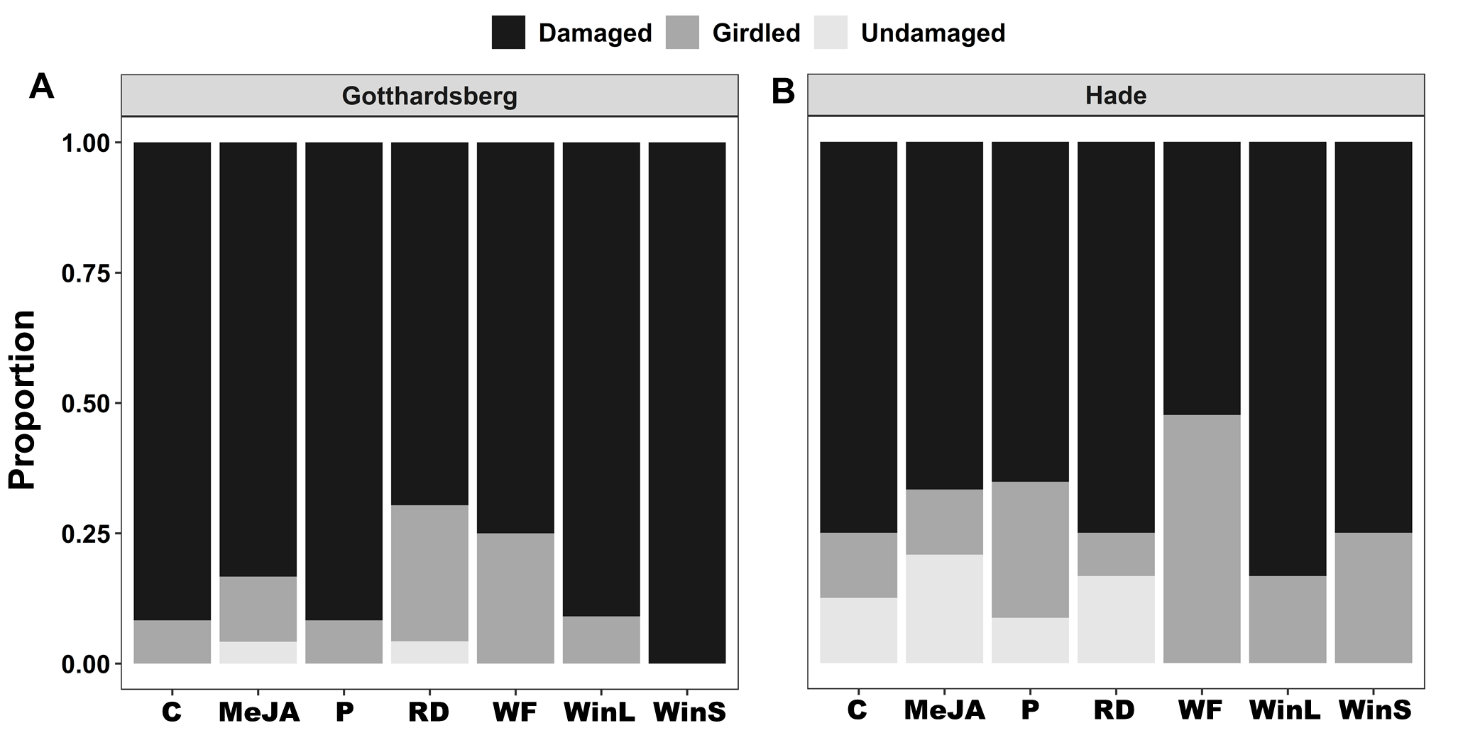
**Supplementary Figure 2.** Proportion of Scots pine (*P. sylvestris*) seedlings receiving different plant defense induction treatments (Undamaged seedlings as controls (C), 10 mM MeJA (MeJA), needle-piercing damage to the stem bark (P), root bark damage (RD), previous weevil feeding damage (WF), large stem window damage (WinL), and small stem window damage (WinS)) that were undamaged, damaged and girdled for two provenances during experiment 1 (insect feeding tests were conducted at one time point, 12 days post-treatment). **(A)** Provenance Gotthardsberg and **(B)** Hade. “Damaged” refers to seedlings that received pine weevil damage but were not girdled during the feeding test; “Girdled” refers to seedlings for which an entire ring of stem bark around the circumference was removed by the pine weevil; “Undamaged” refers to seedlings that did not receive any pine weevil damage during the feeding test.


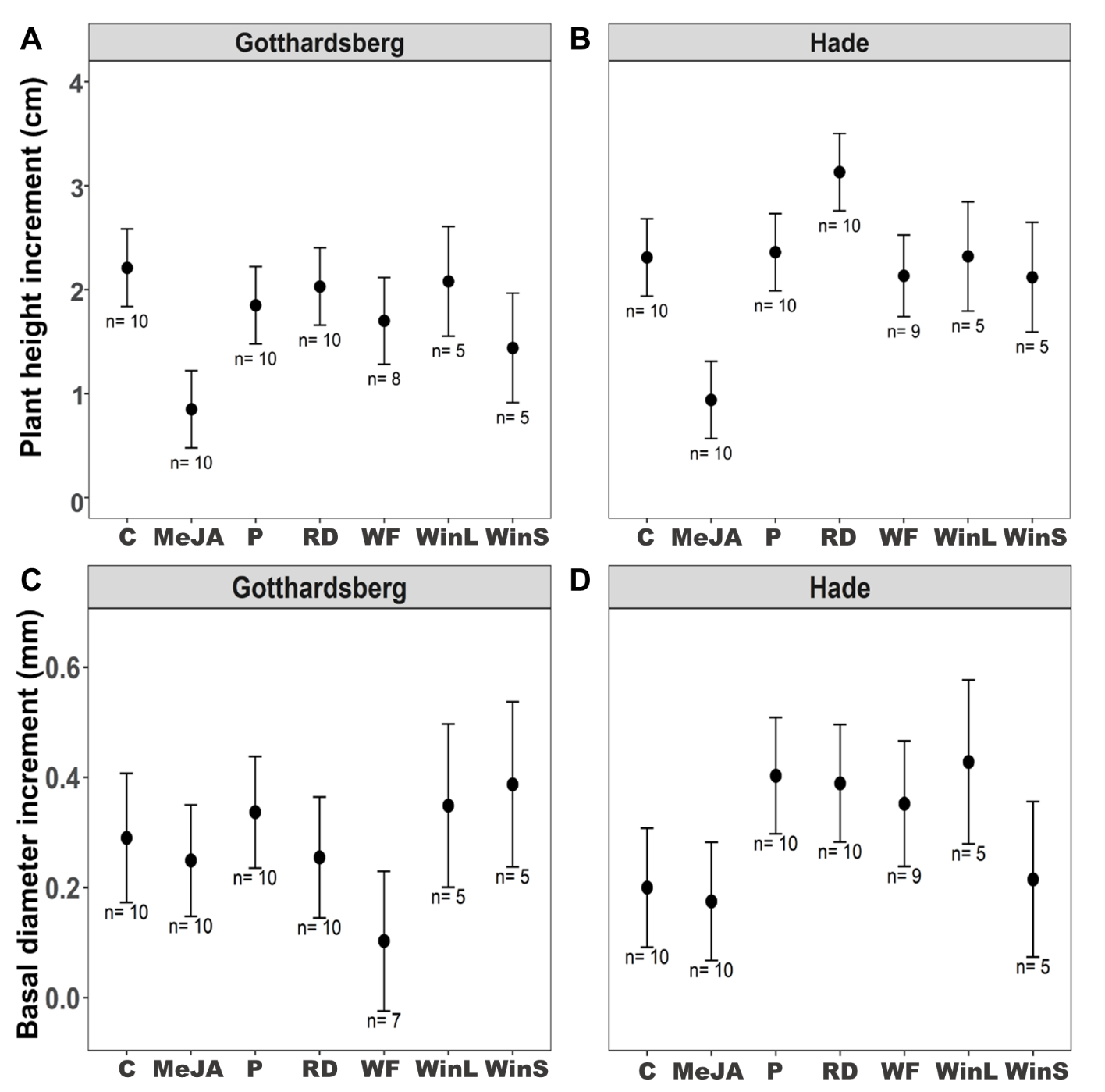
**Supplementary Figure 3.** Estimated mean height increment (cm ± 95% confidence intervals) and diameter increment (mm ± 95% confidence intervals) for two provenances of Scots pine (*P. sylvestris*) seedlings receiving different plant defense induction treatments (Undamaged seedlings as controls (C), 10 mM MeJA (MeJA), needle-piercing damage to the stem bark (P), root bark damage (RD), previous weevil feeding damage (WF), large stem window damage (WinL), and small stem window damage (WinS)) in experiment 1 (Insect feeding tests were conducted at one time point, 12 days post-treatment). Panels **(A)** and **(C)** show provenance Gotthardsberg, plant height and diameter; panels **(B)** and **(D)** show provenance Hade, plant height and diameter. The growth of seedlings was followed for 21 days post-treatment. Sample sizes (n) used in the statistical analyses are also shown.


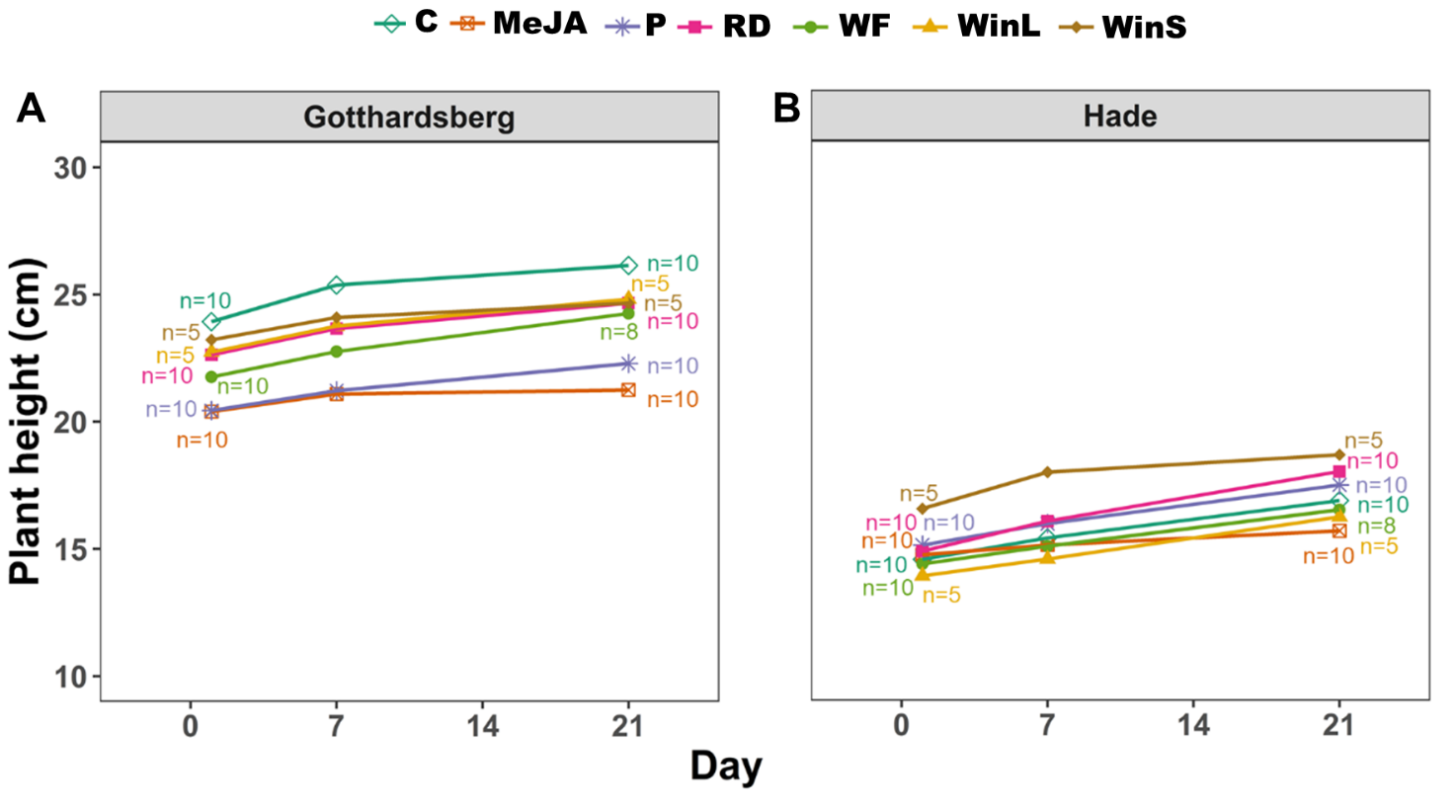
**Supplementary Figure 4.** Height of Scots pine (*P. sylvestris*) seedlings receiving different plant defense induction treatments (Undamaged seedlings as controls (C), 10 mM MeJA (MeJA), needle-piercing damage to the stem bark (P), root bark damage (RD), previous weevil feeding damage (WF), large stem window damage (WinL), and small stem window damage (WinS)) in experiment 1 (Insect feeding tests were conducted at one time point, 12 days post-treatment). **(A)** Provenance Gotthardsberg and **(B)** Hade. Weekly measurements were conducted over 21 days, but data for day 14 is missing.


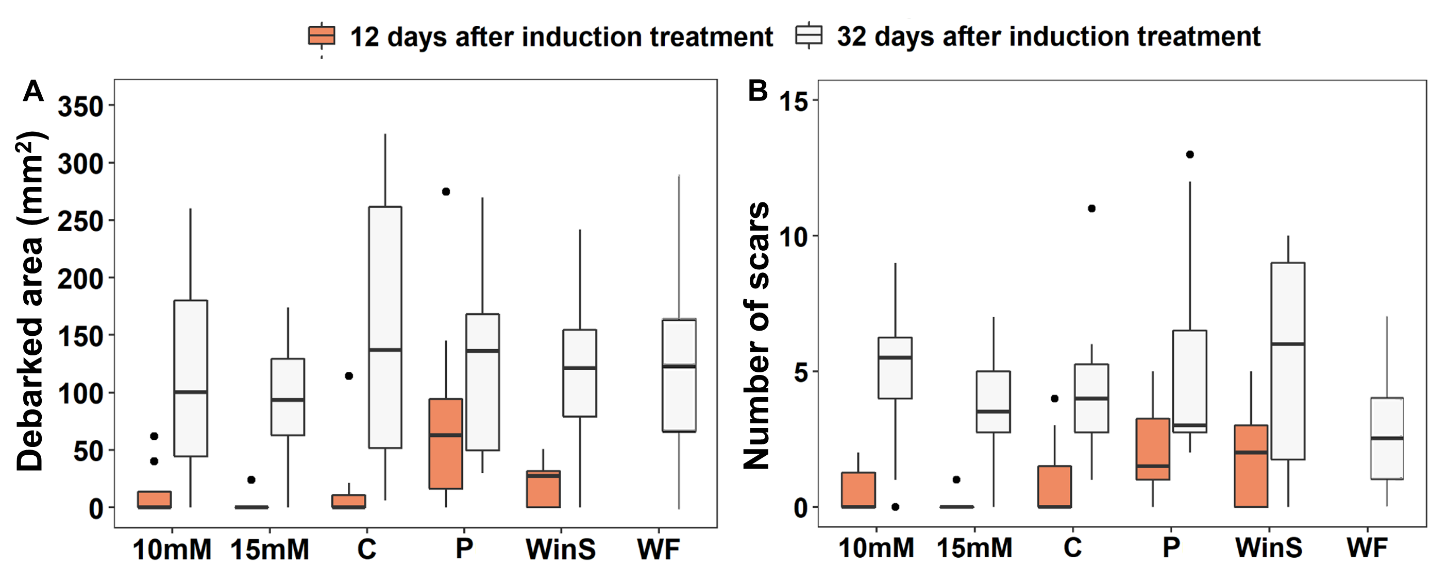


**Supplementary Figure 5.** Area debarked by pine weevils and number of scars on the stem of Scots pine seedlings in experiment 2 (Insect feeding tests were conducted at two time points, 12 and 32 days post-treatment). Plants that were exposed to pine weevils 12 days after induction treatments (10 mM MeJA, 15 mM MeJA, undamaged seedlings as controls (C), needle-piercing damage to the stem bark (P), small stem window damage (WinS), and previous weevil feeding damage (WF) (not included in the weevil exposure test conducted 12 days post-treatment)) are referred to as early exposure, and those that were exposed 32 days after, are referred to as late exposure in the main text. **(A)** Boxplot for debarked area (mm^2^) (raw data), and **(B)** Boxplot for number of feeding scars (raw data). Ends of lines represent the sample minimums and maximums, the boxes represent the lower and upper quartiles, the solid black line is the median, and the solid dots represent potential outliers.


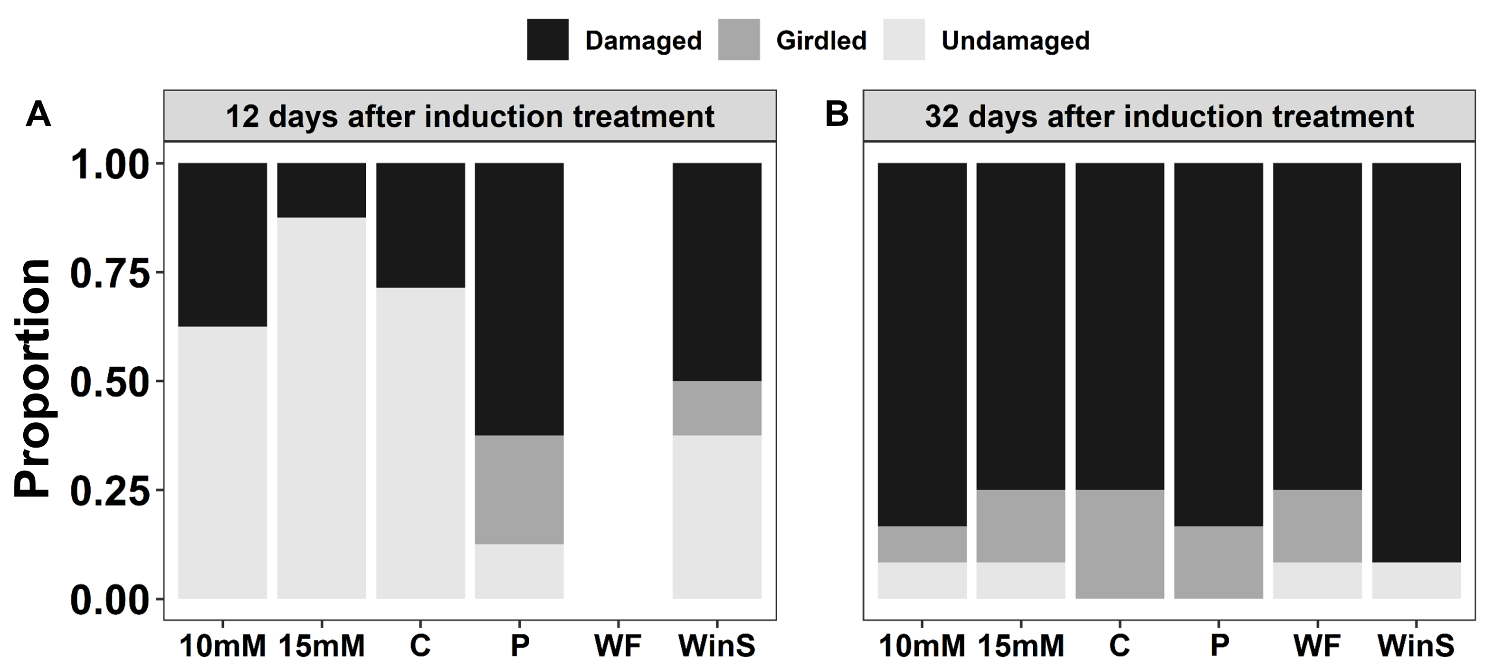


**Supplementary Figure 6.** Proportion of Scots pine (*P. sylvestris*) seedlings receiving different plant defense induction treatments (10 mM MeJA, 15 mM MeJA, undamaged seedlings as controls (C), needle-piercing damage to the stem bark (P), small stem window damage (WinS), and previous weevil feeding damage (WF) (not included in the weevil exposure test conducted 12 days post-treatment) that were undamaged, damaged and girdled during experiment 2 (Insect feeding tests were conducted at two time points, 12 and 32 days post-treatment). In panel **(A)** 12 days (n = 8 for all treatments except that n = 7 for the control (C) group) and **(B)** 32 days after treatments were inflicted. Plants that were exposed to pine weevils 12 days after induction treatments are referred to as early exposure, and those that were exposed 32 days after, are referred to as late exposure in the main text. “Damaged” refers to seedlings that received pine weevil damage but were not girdled during the feeding test; “Girdled” refers to seedlings for which an entire ring of stem bark around the circumference was removed by the pine weevil; “Undamaged” refers to seedlings that did not receive any pine weevil damage during the feeding test.


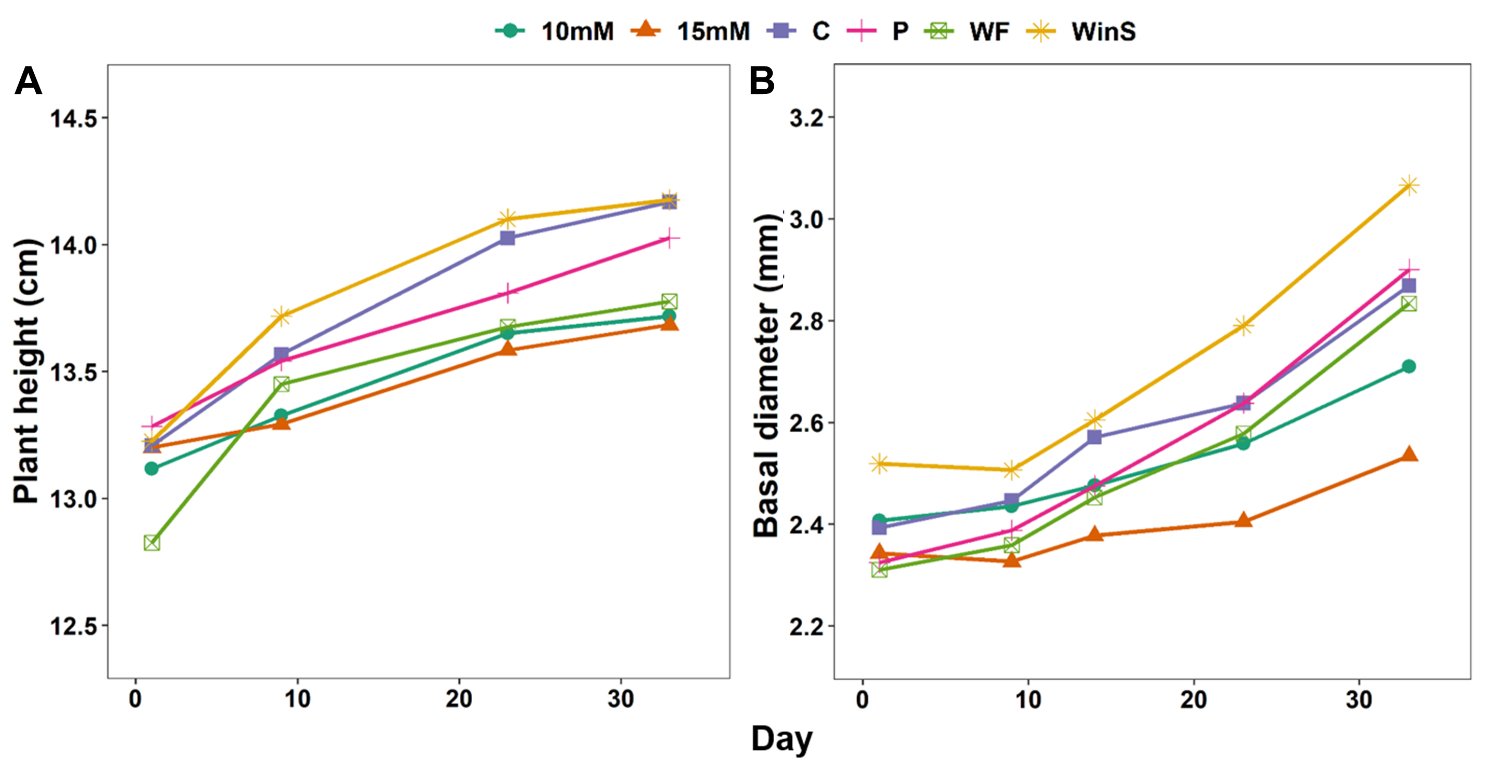


**Supplementary Figure 7.** Seedling height and basal diameter of Scots pine (*P. sylvestris*) seedlings receiving different plant defense induction treatments (10 mM MeJA, 15 mM MeJA, undamaged seedlings as controls (C), needle-piercing damage to the stem bark (P), small stem window damage (WinS), and previous weevil feeding damage (WF)) in experiment 2 (n = 12 per treatment, insect feeding tests were conducted at two time points, 12 and 32 days post-treatment ). **(A)** Plant height (cm) and **(B)** plant diameter (mm).Weekly measurements were conducted over 33 days, but height data for day 14 is missing.


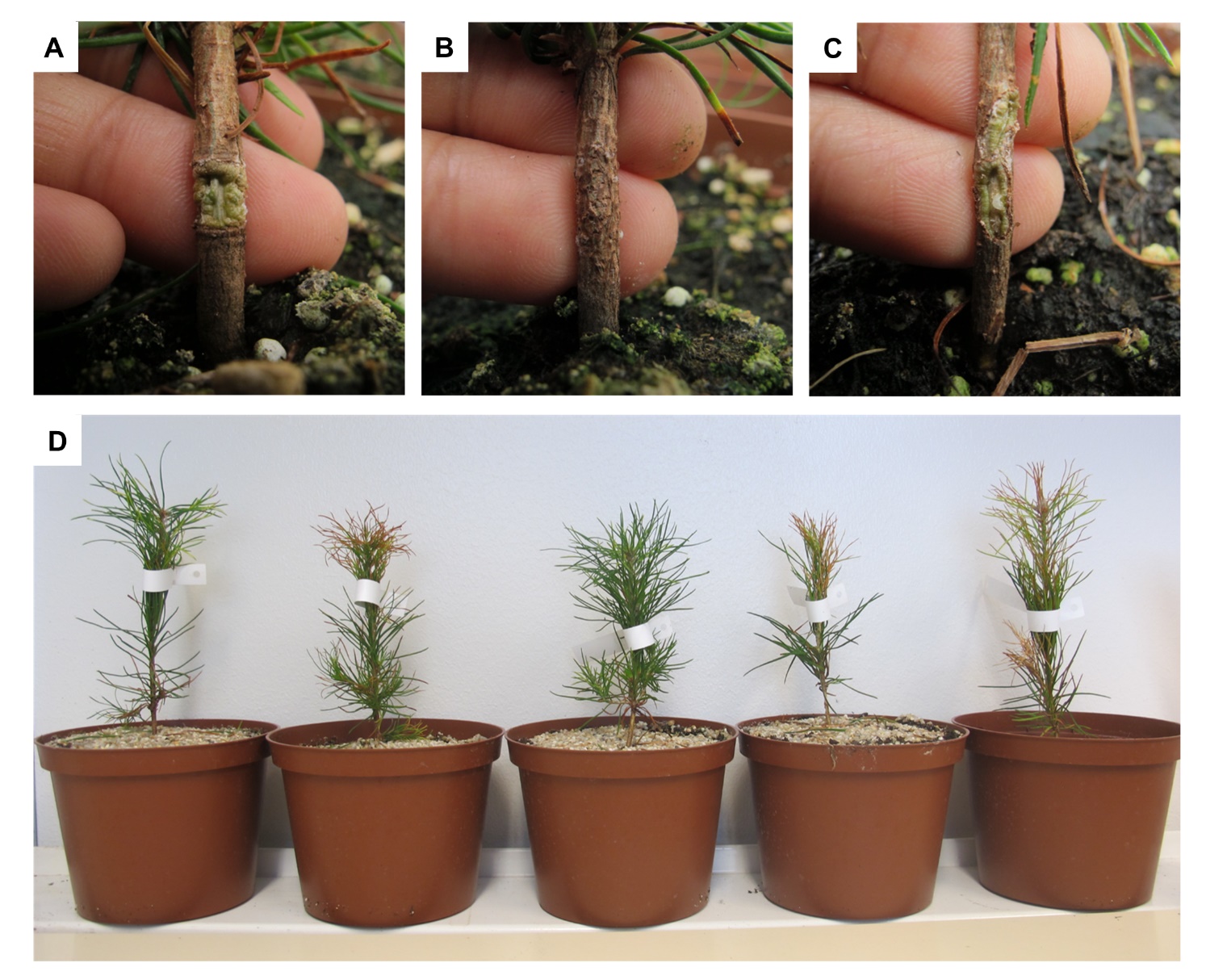


**Supplementary Figure 8.** Pictures of seedlings from experiment 2 (Insect feeding tests were conducted at two time points, 12 and 32 days post-treatment) receiving small stem window damage (WinS), needle-piercing damage to the stem bark (P), previous pine weevil damage (WF) and 15 mM MeJA, 32 days after defense induction treatments occurred, which is referred to as the late exposure group in the main text. **(A)** WinS; **(B)** Piercing; **(C)** WF; and **(D)** 5 seedlings from the 15mM MeJA group showing that a few of them exhibited needle-browning at the top and on side branches.
